# Massive Pulmonary Embolism as a Cause of Cardiac Arrest: Navigating Unknowns in Life After Death

**DOI:** 10.7759/cureus.8361

**Published:** 2020-05-30

**Authors:** Robin Mata, Gabrielle McDermott, Lorenzo Diaz

**Affiliations:** 1 Internal Medicine, Nova Southeastern University School of Osteopathic Medicine, Fort Lauderdale, USA; 2 Physical Medicine and Rehabilitation, Memorial Regional Hospital / Nova Southeastern University-Dr. Kiran C. Patel College of Osteopathic Medicine (KPCOM), Hollywood, USA

**Keywords:** inpatient care, va-ecmo, massive pulmonary embolism, thrombectomy, alteplase, tpa, pert, pulmonary critical care, acute pulmonary embolism, systemic thrombolysis

## Abstract

Pulmonary embolism (PE) is a common diagnosis with a low associated mortality rate. More critical variants, such as massive PE, also known as fulminant PE, are characterized by severe hemodynamic instability and have a markedly higher mortality rate. These variants can later develop in previously low to intermediate-risk patients and precipitate cardiac arrest within hours of symptom onset. The high mortality rate associated with massive PE is confounded by the difficulty in identifying patients most at risk of decompensating and a lack of clear treatment guidelines. We present the case of a patient at low to intermediate-high risk upon admission, and after failing systemic thrombolysis, decompensated, and went into cardiac arrest. This article serves to reinforce the need to closely monitor these patients due to the insufficiency of prognostic scores to predict decompensation and highlights the need for further research. We advocate the use of venoarterial extracorporeal membrane oxygenation (VA-ECMO) as means of stabilization and will discuss various therapeutic alternatives.

## Introduction

Pulmonary embolism (PE) is a common diagnosis, with an associated mortality of 14.7% in those receiving treatment [1]. The initial presentation of PE varies and requires efficient evaluation to reduce morbidity and mortality. Well-validated risk scores such as the Pulmonary Embolism Severity Index (PESI) and American Heart Association (AHA) guidelines which include imaging evidence of right ventricle (RV) strain or cardiac biomarkers may be used to estimate short-term mortality [2,3]. Special consideration should be taken for patients categorized as submassive or intermediate-risk under AHA guidelines, which is defined as hemodynamically stable PE with RV strain. Although these patients may have low-risk scores upon admission they warrant recurrent risk evaluation for the development of massive PE, also known as fulminant PE. Massive PE, which is characterized by severe hemodynamic instability, can later develop and potentiate cardiac arrest within 1-2 hours of onset [4]. Patients who progress to cardiac arrest have associated mortality of 65-95% [5].

The current recommended treatment for acute PE is the continuous infusion of 100 mg of alteplase (tPA) over two hours. However, in cases of hemodynamic instability, bleeding risk, or refractory clots, there are no set protocols [6]. This presents a series of challenges for physicians as they consider the interventional treatments that balance patient risks and benefits. Because pulmonary mainstream obstruction leads to increased RV afterload and failure, most patients will require cardiopulmonary resuscitation (CPR) before they can receive treatment [7]. Not all patients will achieve a return of spontaneous circulation with CPR, meaning alternative methods such as venoarterial extracorporeal membrane oxygenation (VA-ECMO) will be required for stabilization.

VA-ECMO is a unique form of cardiopulmonary bypass developed in the 1970s and was originally used only in select temporary bypass scenarios [8]. Over the past decade, its use has become more widespread as a bridge to medical therapy in cases of cardiac and pulmonary failure such as massive PE [9]. This diversified use has helped decrease mortality, unload RV strain to allow for cardiac recovery, and afford additional time to assess multiple therapeutic interventions [8].

## Case presentation

**Figure 1:**
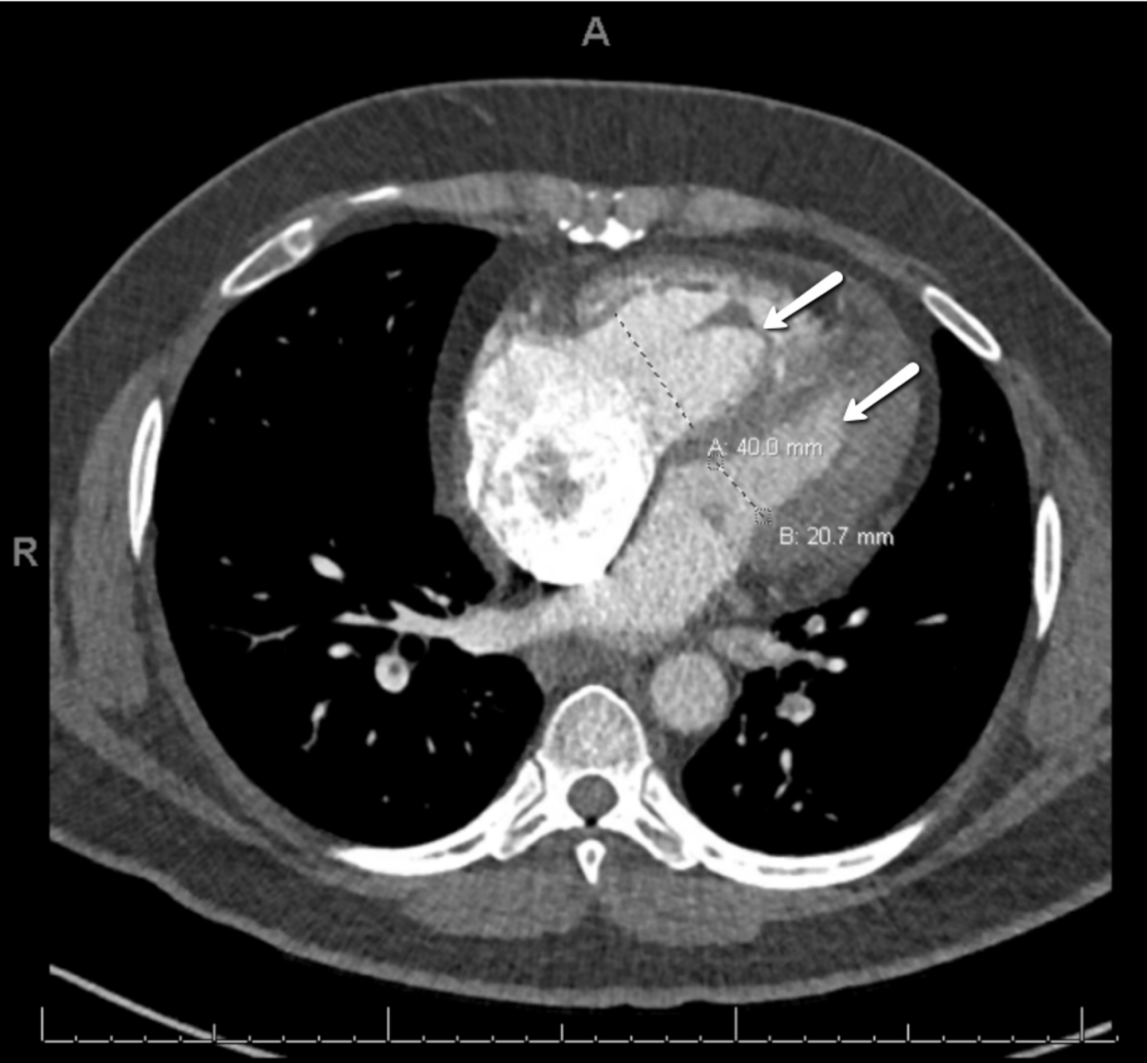
CT. Dilation of the right ventricle (40 mm) relative to the left ventricle (20.7 mm). The ratio of 2:1 suggests right heart strain.

**Figure 2:**
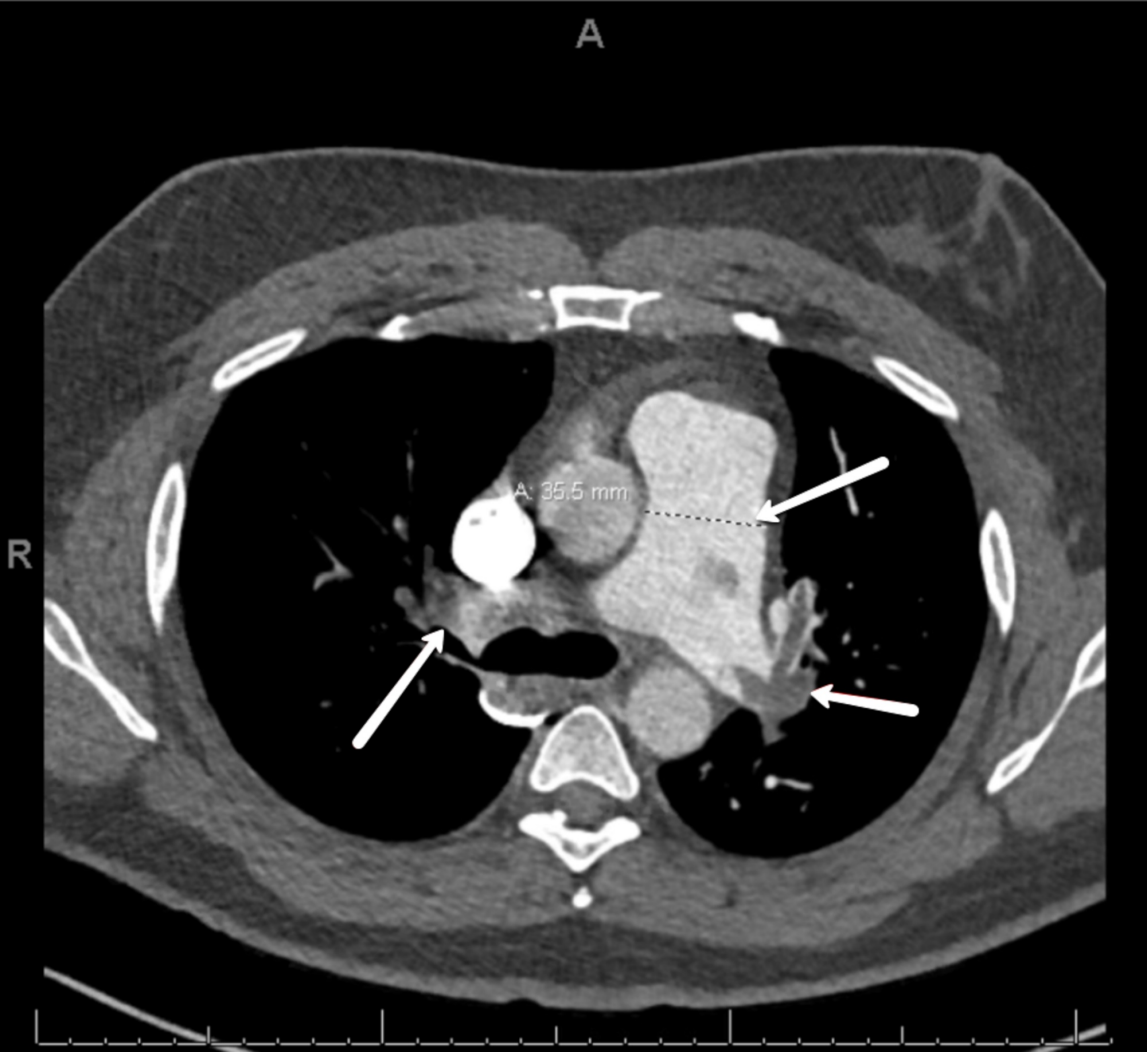
CT. Obstruction of right and left pulmonary arteries by thrombi. Dilation of the pulmonary artery to 35.5 mm.

A 42-year-old male with progressive shortness of breath of four days duration was brought to the hospital by his wife after experiencing a syncopal episode. The patient was employed as a taxi driver and had no other significant medical history or recent trauma. On physical examination, the patient was alert, oriented, and without signs of heart failure, a blood pressure of 139/96 mm Hg, heart rate of 113 bpm, and respiratory rate of 22 bpm. His oxygen saturation was 94% on room air and temperature was 97.4°F. No neurological deficits were appreciated and computed tomography (CT) of the head showed no intracranial pathology. Workup revealed significant bilateral pulmonary artery emboli, and right ventricular strain on CT of the chest (Figures 1, 2), as well as right ventricular emboli and reduced systolic dysfunction on transesophageal echocardiogram (TEE) and pro-brain natriuretic peptide (BNP) of 1,621 pg/mL. This patient’s presentation conferred class II low-risk PESI score of 72. After taking into account the substantial clot burden and right heart strain, the patient was admitted for monitoring. A heparin drip was initiated and the PE response team was consulted immediately.

Twelve hours after admission the patient was experiencing progressive and unresolving tachypnea, prompting transfer to the cardiac intensive care unit (ICU) where contraindications to systemic thrombolysis were ruled out and a continuous infusion of tPA was initiated. Within one hour of beginning the infusion, the patient complained of headache, raising concern for intracranial hemorrhage. The infusion was stopped and the patient was taken for a CT of the head. During transfer the patient became hypotensive and went into cardiac arrest. After multiple failed resuscitation attempts, VA-ECMO was initiated to stabilize him. Once stable, multiple therapy options were considered by the team, but ultimately, percutaneous mechanical thrombectomy was implemented as it best maximized clot removal and minimized mortality risk in this patient. Late complications throughout the hospital course included pericardial effusion, infarct cerebrovascular accident, and acute kidney injury. Despite these sequelae, the patient was able to recover and successfully completed rehab therapy.

## Discussion

Based on the patient’s initial presentation and vitals, the PESI score of 72 conferred a class II-relatively low risk of 1.7-3.5% 30-day mortality, indicating outpatient management. Under AHA guidelines, imaging evidence of RV strain and elevated cardiac biomarkers places the patient in an intermediate-high risk category [10]. The pulmonary embolism thrombolysis (PEITHO) trial estimates that approximately 10% of initially normotensive patients with PE and evidence of RV strain (intermediate-high risk) will decompensate hemodynamically (massive PE) and suffer high mortality [11]. Despite the high mortality associated with these conditions, available data does not support the routine use of intervention, even though some patients with submassive PE will require urgent therapy for stabilization [10]. This case highlights the need for close monitoring due to the insufficiency of current prognostic prediction scores to predict short-term outcomes in isolated patients, advocates for the use of VA-ECMO to decrease mortality in patients with massive PE and defines a clear need for further research.

VA-ECMO is useful in life-threatening cases as it can provide oxygenation, unload RV strain for cardiac recovery, and afford additional time to weigh risks/benefits of treatment. Several small studies have retrospectively shown the use of VA-ECMO in massive PE as salvage intervention and one prospective study used a protocolized approach to initiate VA-ECMO early on in patients who showed signs of end-organ dysfunction or unclear neurologic status. These studies reported overall survival ranging from 60 to 95%, with varying success depending on patient selection, therapeutic intervention chosen for clot removal, and whether treatment was performed before or after initiating VA-ECMO [12-15]. Although no definitive conclusions can be made, these studies suggest that the early use of VA-ECMO may be beneficial in appropriately selected patients [12].

Several treatment modalities can recanalize occluded pulmonary arteries including systemic anticoagulation (such as heparin), systemic thrombolysis (such as tPA), catheter-directed fibrinolysis (with or without ultrasound guidance), percutaneous thrombectomy, and surgical pulmonary embolectomy. Each option carries inherent risks and benefits, ranging from treatment failure and reduced clot removal to major bleeding and increased mortality or long-term complications. Currently, treatment modalities are selected on a case by case basis. In this case, the patient had previously failed both anti-coagulation and systemic thrombolysis. In this patient who had a suspected clot in transit from the RV, catheter-directed thrombolysis raised the risk of embolization. Taking into account the bleeding risk and increased mortality with surgical embolectomy, percutaneous mechanical thrombectomy was chosen as it maximized clot removal and minimized mortality.

The paucity of reliable data underlies the need for large scale randomized controlled trials for the use of VA-ECMO and interventional therapies in massive PE, with the main objective of establishing a patient identification criteria and developing a standardized treatment protocol for combining VA-ECMO with interventional treatment. Based on this case, variables that could be investigated include the effect of VA-ECMO and interventional therapies on long-term complications, optimal duration on VA-ECMO, the timing of interventional treatments, and extent of clot removal achieved by each modality.

## Conclusions

This case demonstrates a patient with low to intermediate-high risk prognostic scores upon admission who developed massive PE. The patient survived cardiac arrest due to the use of VA-ECMO and was treated with percutaneous mechanical thrombectomy. VA-ECMO was useful in unloading right ventricle strain, allowed for cardiac recovery, and allowed additional time to evaluate therapeutic approaches. Although many diagnostic tools enhance our prognostication in PE, there are still clear gaps in identifying patients most at risk of decompensating. Further studies focused on identifying patients at risk of decompensation, optimizing VA-ECMO settings, and duration of use, as well as comparing treatment efficacy, will help us better address how to best reduce mortality and long-term complications in patients with massive PE.
